# Ability grouping in German secondary schools: The effect of non‐academic track schools on the development of Math competencies

**DOI:** 10.1111/bjep.12741

**Published:** 2025-02-14

**Authors:** Sonja Herrmann, Katharina M. Bach

**Affiliations:** ^1^ German Youth Institute Munich Germany; ^2^ Faculty of Psychology and Educational Sciences Ludwig‐Maximilians‐Universität München Munich Germany

**Keywords:** ability grouping, competence development, math literacy, propensity score analysis, secondary school

## Abstract

**Background:**

Differences in competence gains between academic and non‐academic track schools are often attributed to selection effects based on students' primary school performance and socioeconomic status (SES). However, how the competencies of comparable students (in terms of school performance and social background) at different tracks develop is often neglected.

**Aims:**

We investigated whether comparable students diverge in their math competencies due to attending different types of secondary schools, contributing to the ongoing debate on whether inaccurate stratification may lead to disadvantages.

**Methods:**

Using data from the National Education Panel Study (Kindergarten Cohort SC2, *N* = 4180), we examined students' competence development from the fourth to seventh grade.

We employed a quasi‐experimental design (propensity score weighting, PSW) comparing similarly capable students at academic and non‐academic school tracks to make causal inferences. The outcome variable was students' math competence in seventh grade. PSW used fourth‐grade competency measures in math and reading and other variables such as sex, migration background, SES, class composition, special educational needs, school grades and school location.

**Results:**

Results revealed a significant average treatment effect on the treated, indicating that comparable students attending non‐academic track schools show lower math performance than those at academic track schools.

**Conclusion:**

Non‐academic tracks seem to hinder the full development of students' competencies. We conclude that the effects of preconditions like the students' SES, ability and aspirations on competence development are lower than assumed and that school learning environments should be given greater importance. We discuss practical solutions and provide suggestions for future research.

## INTRODUCTION

Unlike systems in Anglo‐Saxon regions, where ability grouping occurs within the schools, in the German school system, students are sorted into different types of schools that differ in overall instruction, curricula and equipment (Baumert et al., 2010; Esser & Relikowski, 2015; Guill et al., 2017; Kunter et al., 2005; Pribesh et al., 2011). More precisely, they transfer to either academic or non‐academic schools after elementary school based on their primary school performance (Cortina et al., 2008; Faust, 2006). The aim of grouping students into homogeneous ability groups is to offer optimal support according to their needs by tailoring instruction, materials and pace to the prerequisites and levels of the specific group (Hattie, 2002). Past research results regarding ability grouping are, however, ambiguous: While various studies point to a disadvantage of students with lower socioeconomic status (SES) and an increase in social inequality (so‐called ‘scissor effect’, e.g., Ditton et al., 2005; Maaz, Neumann, et al., 2008; Scheeren, 2022), some research suggests benefits of tracking, especially for gifted students (e.g., Esser & Relikowski, 2015). Overall, most studies still agree that across different subjects, students in academic track schools in particular benefit from tracking, while it is often disadvantageous for students in non‐academic track schools (e.g., Angelone, 2019; Becker et al., 2014; Köller et al., 2013; Köller & Baumert, 2001; Scharenberg, 2014; Traini et al., 2021).

Assignment to non‐academic track schools appears disadvantageous for students, especially when they qualify for academic track schools. These incorrect assignments can have long‐term adverse effects due to limited upward permeability between tracks. However, there is little research on the impact of these incorrect assignments on students' performance development. Our study examines the development of math competences of students in primary school who were—especially with regard to their Math performance—suitable for academic track schools but placed in non‐academic track schools. We aim to determine whether these students fall behind their classmates who transferred to an academic track school or can maintain their performance levels while attending a non‐academic track school.

### Tracking in the German school system

Vertical segmentation in secondary education, where students are placed in different school types or tracks based on academic performance, aims to cater to students' diverse needs and improve teaching efficiency (e.g., Ariga & Brunello, 2007; Hattie, 2002). The assumption is that students have different educational needs (Bloom, 1976), which can best be met by teaching homogeneous classes. This separation occurs in Germany very early after the fourth grade, approximately at the age of ten (Hanushek & Wößmann, 2006).^1^ Early tracking is, therefore, often discussed as a decisive factor for educational inequality in Germany (Neugebauer & Schindler, 2012; Terrin & Triventi, 2023).

The German school system has a tripartite structure, with two main groups that primarily differ regarding their orientation, the length of schooling and the school‐leaving certificates. Academic track schools that prepare students for university (*Gymnasium*) and non‐academic track schools that focus on vocational apprenticeship (*Real‐ and Hauptschule*; Becker et al., 2012). Gymnasium, the most rigorous track, spans eight to nine years, and students graduate with the *Abitur*, which is a requirement to enter university, followed by Realschule with an intermediate level (6 years), and Hauptschule, the shortest and least demanding type (5–6 years; Hosenfeld et al., 1999). Since the 1970s, comprehensive schools (*Gesamtschulen*) in several federal states allow students to attend regardless of performance. Different ability groups exist within these schools, enabling students to choose various tracks and graduate with different qualifications (Helbig & Nikolai, 2015). However, we do not consider these schools in the present study.

Academic achievement in elementary school largely determines placement in specific types of secondary schools (e.g., Esser & Relikowski, 2015). However, factors like migration background and SES influence decisions (e.g., Maaz, Trautwein, et al., 2008; Pfost & Artelt, 2013). Depending on the federal state, teachers recommend the type of school each student should attend, either binding or non‐binding.^2^ These recommendations do not simply rely on students' performance but are frequently biased by teachers expectations, which might be affected by students' SES (Thys, 2018). Different school types offer distinct learning environments, shaping students' development opportunities differently. Specifically, they diverge in their educational objectives for students, teaching culture, curricula and teacher training (Neumann et al., 2007). In particular, the fact that specific school‐leaving certificates such as the *Abitur* can only be obtained at academic track schools but are necessary to pursue more prestigious career paths or higher education shows what an impact the change to secondary school can have (Dumont et al., 2013).

#### Selection into tracks

As described above, selecting school types is mostly but not solely based on performance (van Leest et al., 2021). Additionally, subjective factors can influence teachers' suggestions, leading to a structural disadvantage for particular groups (Batruch et al., 2019). More precisely, children with higher SES have significantly better chances of attending an academic track school than children from working‐class families (Baumert & Köller, 2005). This effect persists even when controlling for individual competencies (Baumert et al., 2001; Ditton et al., 2005). Other factors, including migration history and sex, also exert influence (Connolly et al., 2019). For example, girls have a slightly higher chance of being placed in an academic track than boys (Ditton & Krüsken, 2006; Gamoran & Mare, 1989). Students with migration backgrounds are not only often graded worse (Bonefeld & Dickhäuser, 2018), more frequently leading to recommendations for non‐academic track schools but, despite similar grades and abilities, less often recommended for academic track schools compared to their peers without migration background (Lüdemann & Schwerdt, 2013).

A similar pattern can be found in parental educational choices for their children since they often make the final decision. These decisions frequently depend on the parents' academic aspirations, which are usually related to their SES (i.e., the highest level of parental education and profession). In particular, the father's working environment and associated social relationships have a crucial effect on the type of secondary school parents choose for their children (Dustmann, 2004). Moreover, past research highlighted that despite equal performance in elementary school, parents with higher SES are more likely to transfer their children to academic track schools even if their grades indicated a better fit for a non‐academic track school (Dumont et al., 2013). Thus, the intention of ability grouping to create more homogeneous schools is only partially achieved because many factors unrelated to ability affect the tracking (Valtin, 2005).

Another significant issue associated with the selection into tracks is the limited permeability between school types later (Valtin, 2005). Changing schools after the initial placement is typically challenging, and upward change in tracks happens less often than vice versa (Blossfeld, 2018; Hoffer, 1992; Lauterbach & Fend, 2016). Students might remain in a school that is not appropriate for them, which will have a long‐term impact on their academic achievements and future opportunities (Baumert et al., 2001). Societal stigma often surrounds non‐academic track schools, affecting students' motivation and teachers' engagement at non‐academic track schools even further (Hattie, 2002).

#### Consequences of track attendance

The debate about the consequences of tracking remains controversial. Proponents of tracking highlight benefits in homogeneous student compositions (Esser & Relikowski, 2015). Instruction and pace can be tailored to the needs of more homogenous students, aiming to prevent students from feeling overwhelmed or under‐challenged. Positive outcomes of tracking include enhanced academic performance and positive development of personality and social skills (Neuenschwander et al., 2012). High‐achieving students in academic tracks tend to benefit (Arbeitsgruppe Bildungsbericht, 1994; Argys et al., 1996; Card & Giuliano, 2016). Critics argue that the early segregation into different school types contributes to social disparities among students and, thus, fosters the educational gap (e.g., Hanushek & Wößmann, 2006; Reichelt et al., 2019). Graduation rates and job‐market opportunities (e.g., fewer vocational choices) further reflect disparities between students attending different tracks, building unequal foundations for students' futures and socioeconomic development (e.g., Domina et al., 2017; Gamoran & Mare, 1989).

Academic track schools tend to offer higher quality education, resulting in advantages for students' personal development (Becker et al., 2012; Guill et al., 2017), academic achievement (Neuenschwander et al., 2012) and career opportunities (Haeberlin et al., 2004). Students in non‐academic track schools often lack these benefits and are disadvantaged due to the quality disparities between the different schools (Hoffer, 1992). Early research demonstrated differences in academic achievement between students in academic‐ and non‐academic track schools, with students in academic track schools outperforming their peers (Hosenfeld et al., 1999).

#### Compositional and institutional effects explaining the consequences of tracking

According to Baumert et al. (2006), compositional and institutional effects account for differences among students attending different school types. Academic track and non‐academic track schools often differ in the composition of the students. As the description of the tracking decisions above pointed out, the groups ultimately vary not only based on their abilities and performance but also regarding their social and cultural characteristics. Academic track schools often enroll students with higher SES (e.g., Dumont et al., 2013; Esser & Relikowski, 2015). In contrast, non‐academic track schools typically have more students with various risk factors related to family characteristics, such as lower SES (Neumann et al., 2007), and significantly more students with a migration background (Kristen, 2002). While lower SES does not equate to low performance, there is still a strong influence of SES on learning‐relevant cognitive (e.g., attention; Hoyer et al., 2023) and motivational‐affective characteristics (e.g., academic self‐concept; Gujare & Tiwari, 2016). These effects are often mediated by a lack of support at home or less favourable home learning environments (e.g., Banerjee, 2016; Hofer et al., 2023). The typical student body at a non‐academic track school can negatively affect other students' achievement (Göllner et al., 2018; van Ewijk & Sleegers, 2010). The absence of diverse abilities might deprive weaker students of the motivation and support otherwise offered by stronger peers (e.g., Biedermann et al., 2015; Hanushek & Wößmann, 2006). So, placement in a lower‐ability group seems to diminish learning effects, while being placed in a high‐achieving group fosters academic improvement due to the spill‐over effects (Becker et al., 2022; Condron, 2008).

Academic track schools tend to outperform non‐academic track schools with regard to teachers' qualifications (Baumert et al., 2010), equipment (Esser & Relikowski, 2015), and quality of instruction (Guill et al., 2017). In Germany, a distinction is made in teacher training between focusing on theoretical and scientific approaches or practical teaching styles depending on which school track the student teachers will be teaching in the future (Baumert et al., 2010). Teachers for academic track schools often have a higher level of specialist knowledge, which they transfer to their students (Ohle & Fischer, 2012). Moreover, the curricula of academic track schools are designed to prepare students for university, while non‐academic track schools are oriented towards practically relevant content and occupation‐specific aspects (Esser & Relikowski, 2015; Kunter et al., 2005). Generally, students at academic track schools can receive academic support at a high level and go into greater depth on certain subjects.

#### Research on the influence of tracking on development in math

The differences in students' performance at academic track schools compared to non‐academic track schools are particularly pronounced for math (Ireson, Clark, & Hallam, 2002). Findings indicate that academic track students achieve significantly higher performance levels than their peers (Guill et al., 2017) and show a stronger development of skills (e.g., Argys et al., 1996). Significant performance differences were also found in standardized tests like PISA (Frey et al., 2010). The divergence in math performance extends over time, indicating a greater increase in achievement at academic track schools than at non‐academic track schools (Pekrun et al., 2006). Investigating similar Swiss students and their math performance in the later course of different secondary levels confirmed widening performance gaps in math due to school‐type‐specific effects (Angelone, 2019). Comparable effects were also found for within‐school grouping in the United Kingdom: Students with initially similar educational performances performed worse in math when sorted into medium‐ or low‐ability groups than those placed in high groups (Ireson, Hallam, et al., 2002).

### Present research and research question

Previous research suggested stronger competence gains in academic track schools compared to non‐academic track schools. These differences are often attributed to initial performance in primary school and students' SES (e.g., Maaz, Trautwein, et al., 2008). Research gaps remain in understanding the outcomes of students eligible for academic track schools but attend non‐academic track schools. Do these students fall behind comparable classmates who transferred to an academic track secondary school, or can they maintain their level at the non‐academic track school? Answering this question might shed light on how inaccurate stratification can lead to social disadvantages.

Prior research using quasi‐experimental designs including large datasets exists for Germany (Traini et al., 2021), the US‐American (Slavin, 1990) and Swiss school systems (Angelone, 2019). While the Swiss system is similar to the German system in that it is divided into different types of schools (albeit at a slightly later stage), there is more differentiation within than between schools in the USA. In addition to the differences in the education systems, the studies are also based on different data. Using countrywide panel data allows us to get a large and representative sample in contrast to Angelone (2019), who applied a similar methodological approach but with a much smaller sample restricted to one specific region of the country only, which they themselves criticized for its lack of generalizability. Moreover, in contrast to Traini et al. (2021), we use panel data that allows us to compare children who had similar math achievements in the last year of primary school before tracking and later after tracking. This is a significant advantage compared to Traini et al. (2021), who used data that only included information from the fifth grade, accordingly after tracking. This information on performance in elementary school is a central covariate in the analyses to ensure that students with similar Math performance before the tracking are compared, making the estimation of the treatment effect more precise. Moreover, by controlling for pre‐existing performance differences, the causal inference regarding the effects of tracking on academic outcomes is strengthened.

By using a different methodological approach, a representative nationwide and large sample, and focusing on Germany, a country which is known for its early and rigid tracking system (Shavit & Müller, 2000; van de Werfhorst & Mijs, 2010), we aim to extend prior research by exploring the academic performance of students with similar math competence transitioning from primary to secondary school. The results will allow us to see whether attending different school tracks (academic vs. non‐academic) affects students' math performance in grade seven. The performance should not differ too much for students with similar basic conditions. If significant differences in math abilities emerge later in secondary school, it is likely due to factors at non‐academic track schools, such as lower‐quality instruction and less effective competency development.

## MATERIALS AND METHODS

### NEPS data and the kindergarten cohort (SC2)

The National Educational Panel Study (NEPS), conducted by the Leibniz Institute for Educational Trajectories, is a longitudinal study designed to advance empirical educational research in Germany. The study aims to track educational processes and educational returns throughout all life stages for students in Germany (Blossfeld et al., 2011). We used the NEPS SC2 data for our analyses, which comprised information on developing competencies from kindergarten to the different secondary school levels. The NEPS study was employed as a two‐stage sampling procedure to cover as many kindergarten children as possible later in primary school. First, primary schools across Germany were stratified according to federal states and sponsorships, soliciting their participation in the NEPS main survey in the 2012/2013 school year. Second, target kindergartens were selected by identifying the provider kindergartens of the participating primary schools.^3^

The children were subject to regular tests to obtain information on competence development and educational pathways. Math tests were administered to students from the first grade onwards. We used the competence measures of the fourth grade (wave 6) and the seventh grade (wave 9) for our analyses. No tests were conducted in the fifth and sixth grades.

### Assessment of Math competencies in NEPS

The math competence tests in NEPS were constructed based on PISA tests and national educational standards (Neumann et al., 2013). The math competencies^4^ in SC2 assessed two key abilities: content areas (i.e., quantity, space, shape, change, relationship, data and chance) and cognitive components (i.e., arguing, communication, modelling, problem‐solving, representing and applying technical skills). Across these two domains, students were asked to solve mathematical problems and answer multiple‐choice questions. The paper‐based test contained 24 items in the fourth grade and 21 items in the seventh grade, with a 30 min time limit for completion. For details on the theoretical framework and sample items, refer to Schnittjer and Duchhardt (2015) or Neumann et al. (2013).

### Weighted maximum likelihood estimates (WLE)

Math competencies were placed on a joint scale to adequately compare individual math competencies across all measurement points. Mathematical competencies were estimated using linked‐item difficulty parameters and were subsequently scaled using partial‐credit models (Masters, 1982). Various analyses based on item response theory (IRT) were performed to evaluate the quality of these competence tests. IRT is a method used to measure a person's psychological characteristics that are not directly measurable. Based on IRT, the probability of a specific response can be modelled as a function of measurable parameters (Rost, 1996). Weighted maximum likelihood estimates (WLEs; Warm, 1989) are point estimates, best representing each participant's competence regarding observed responses only (and the assumed item response model). The uncorrected WLEs are linked to the first measurement point in the last year of kindergarten. They can be used if the research focuses on longitudinal analysis questions, such as competence development because score differences can be interpreted as development trajectories across measurement points (Fischer et al., 2016). We opted to use uncorrected WLEs for our analyses, following this guideline. For information on competence scaling, see Pohl and Carstensen (2013); for detailed information regarding quality analyses, see, for example, Schnittjer and Gerken (2018).

### Propensity score analysis with inverse probability weights

We employed a quasi‐experiment using propensity score weighting (PSW) to examine our research question. PSW is a statistical technique used to adjust for confounding variables in observational studies where random assignment is impossible. First, the probability (propensity score—PS) of an individual being assigned to a particular treatment or group based on observed covariates is estimated. Once these PSs are calculated, individuals are weighted according to the inverse of their scores (inverse probability of treatment weighting—IPTW), allowing for the adjustment of the treatment groups to reflect the characteristics of the entire sample better. This weighting process creates balanced groups across observed characteristics, enabling more accurate causal inferences regarding the impact of the different treatments (Leyrat et al., 2019).

To address our research question, we apply Propensity Score Weighting with Inverse Probability Treatment Weighting (IPTW) in R (R Core Team, 2021), using the function *weightthem* from the *MatchThem* package (Pishgar et al., 2021). A generalized linear model (GLM), specifically a logistic regression, is used to estimate the propensity scores for weighting.

The strong ignorability assumption is an essential requirement for estimating causal effects in observational studies using propensity score methods. It consists of two main conditions: no unmeasured confounders and overlap in the propensity scores between treated and control groups (Rosenbaum & Rubin, 1983). This assumption states that conditional on the observed covariates, the assignment to treatment is independent of potential outcomes. However, in practice, it is difficult to confirm whether this assumption entirely holds, as it is always possible that some relevant confounders remain unmeasured. Despite these limitations, we can mitigate the effects of confounding by carefully selecting and including all relevant observable covariates. However, we cannot entirely eliminate the risk of bias in an observational study (Austin, 2011).

### Variables

**FIGURE A1:**
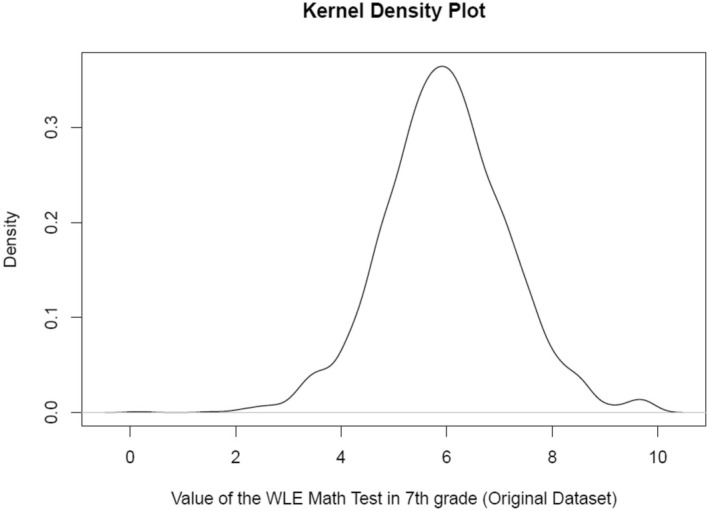
Kernel Density Plot showing the Distribution of the Dependent Variable ‘WLE Math Test 7th Grade’ with the Original Data.

In our study, the outcome variable is the math competence of the target child in the seventh grade of secondary school. The math competence measures are provided as WLEs. WLEs are estimates of a student's most likely competence score (Pohl & Carstensen, 2013). Please see the section ‘Weighted Maximum Likelihood Estimates’ for further information on competence measurement. Please see Figure A1 in the Appendix A for a kernel density plot illustrating the distribution of the WLE for math competence in seventh grade.

As a treatment, we used the school type with the expression ‘academic track school attendance (Gymnasium)’; as the control group, we used ‘non‐academic track school (Real‐ and Hauptschule)’. Due to the unequal sample size, we combine middle and lower non‐academic track schools into one group. As such, 3198 students attend an academic track, and 982 attend a non‐academic track. Special schools (*Förderschulen*) were excluded from the analyses because they are only aimed at students with special educational needs. Furthermore, we excluded comprehensive schools (Gesamtschulen) because it is unclear which track the students attend. The secondary school type attended was the one specified at the time of the competency measurement in seventh grade or the last known date.

For the weighting procedure, we used the fourth‐grade competency measures of the target child in math and reading. In addition, weighting was done on the following variables: grade in math in fourth grade, grade in German in fourth grade, sex of the target child, migration background of the target child, and social status of the parents of the target child via the International Socio‐Economic Index of Occupational Status (ISEI‐88) classification (Ganzeboom et al., 1992). The ISEI‐88 takes values between 16 (e.g., support staff and cleaners) and 90 (e.g., judges). To control for cognitive limitations, we used the variable ‘Special educational needs’ for the analyses. Early or late primary school enrollment can also provide conclusions about children's school performance (Hurrelmann, 1986). In addition, we also checked for a change of school at the secondary level, i.e., a change that took place after the transfer to the secondary level. We also used the secondary school recommendation of the primary school teacher and the realistic and idealistic school qualification aspirations of the student for the analyses.

Furthermore, we used the variable former GDR (East Germany) or FRG (West Germany) for weighting because the social structure still differs between East and West Germany. For example, mothers in East Germany are still more likely to work full‐time, which leads to less involvement in children's education (Barth et al., 2020). Moreover, we used the most important class characteristics for our analyses, such as the share of females (in %), the share of children with at least one parent with a university degree (in %), and the number of math lessons per week. For a detailed description of all variables, see Table 1.

**TABLE 1:** Descriptive statistics of the original dataset *N* = 4180.

	M/percent	*SD*	Min	Max	Mis
WLE math test 4th grade	4.94	1.08	0.03	9.50	9%
WLE reading test 4th grade	0.59	0.22	0.38	2.09	9%
Grade math	2.28	0.87	1	5	15%
Grade german	2.30	0.79	1	5	15%
Special educational needs (Ref. no)	0.97	0.18	0	1	33%
Former GDR (Ref. West Germany)	0.16	0.36	0	1	0%
Enrolment in primary school					33%
Ref. regular	0.91	0.29	0	1	
Early	0.05	0.22	0	1	
Delayed	0.04	0.20	0	1	
Realistic school qualification aspirations					8%
Ref. Non‐academic track (Hauptschule)	0.08	0.27	0	1	
Upper non‐academic track (Realschule)	0.20	0.40	0	1	
Academic track (Abitur)	0.72	0.45	0	1	
Idealistic school qualification aspirations					7%
Ref. Non‐academic track (Hauptschule)	0.05	0.22	0	1	
Upper non‐academic track (Realschule)	0.11	0.32	0	1	
Academic track (Abitur)	0.83	0.37	0	1	
Anticipated secondary school recommendation	0.68	0.47	0	1	25%
Change of secondary school type	0.08	0.27	0	1	0%
Sex (Ref. male)	0.52	0.50	0	1	12%
Migration Background (Ref. none)	0.24	0.43	0	1	15%
SES anchor ISEI‐88	52.72	15.88	16	90	9%
SES partner ISEI‐88	53.41	17.97	16	90	22%
Class: Amount of Math lessons	1.95	0.26	1	3	24%
Class: Share higher‐educated parents	28.44	20.17	0	100	49%
Class: Share females	50.31	9.30	0	100	20%
School type academic track (Ref. Non‐academic track)	0.71	0.45	0	1	0%
Dep. variable					
WLE Math Test 7th grade	5.99	1.19	0.17	9.83	50%

### Multiple imputation with chained equations combining treatment effect estimates (MIte)

We excluded children who had not participated after the fifth wave (third grade) from our analyses. For the remaining participants, we employed multiple imputations with chained equations combining treatment effect estimates (MIte; Leyrat et al., 2019) to combine the treatment effects estimated on each imputed dataset (also called the ‘within’ method). We included the outcome and all weighting variables in the imputation model. Further, all available prior math literacy and reading tests, as well as information on school grades and the parents' educational levels, were included in the imputation model to improve predictive validity. In each case, 20 datasets with 20 iterations each were imputed with classification and regression trees (CART) using the package *mice* (van Buuren & Groothuis‐Oudshoorn, 2011). There were 14% missing values on the fourth‐grade Math competency test and 50% missing values on the seventh‐grade test (see also Table 1). Despite this relatively high number of missings in the dependent variable, we imputed the data instead of conducting a complete case analysis. Simulation studies indicated more accurate estimates after multiple imputations with chained equations, even with many missing values (Lee & Huber, 2021; Raghunathan et al., 2000).

We assume that the data are ‘missing at random’ (MAR), which means that the probability that a value is missing depends on the observed values only and not on the unobserved (Schafer & Graham, 2002). It is plausible to assume this, given that the large proportion of missing data in the seventh‐grade test is mainly due to the survey design and the German school system: The institutional transitions to various secondary schools following the completion of primary school's fourth grade lead to numerous students no longer being involved in follow‐up assessments. Students' dropout at this transition point is inevitable and probably not attributable to a systematic dropout.

Despite students being nested in school classes, using the cluster information in the imputation or error estimation was impossible due to limited observations in some clusters and missing information for some individual‐tracked children. Instead, we used robust Huber–White standard errors in all models, which are heteroskedasticity consistent (Kolenikov & Bollen, 2012).

## RESULTS

**FIGURE 1:**
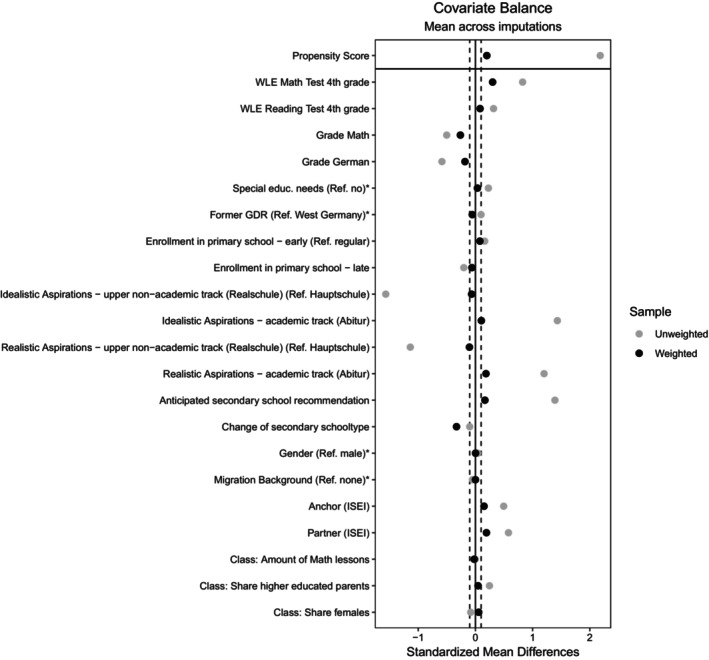
Pooled Mean Differences of all Covariates for the Unweighted and Weighted Sample among Students from Academic and Non‐academic Tracks. *Indicates variables for which the displayed value is the raw (unstandardised) mean difference.

**FIGURE A2:**
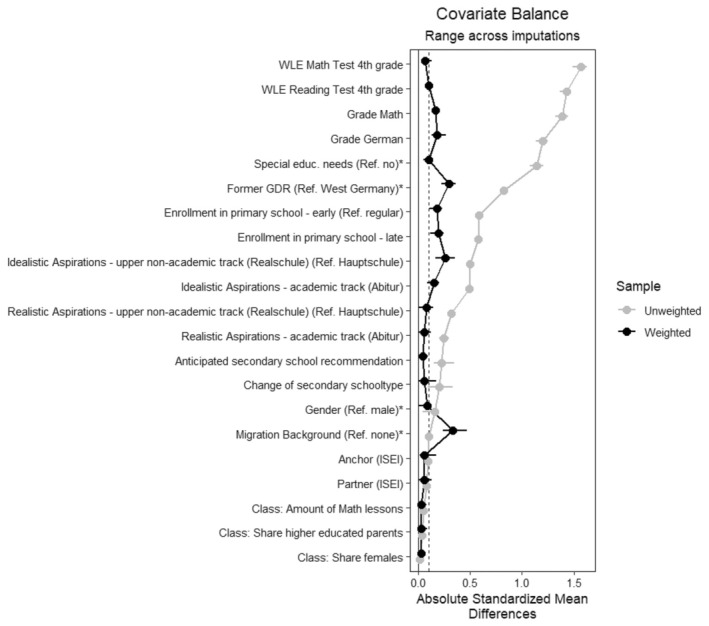
Pooled Absolute Standardized Mean Differences of all Covariates for the Unweighted and Weighted Sample Among Students from Academic and Non‐Academic Tracks. *Indicates variables for which the displayed value is the raw (unstandardised) mean difference.

**FIGURE A3:**
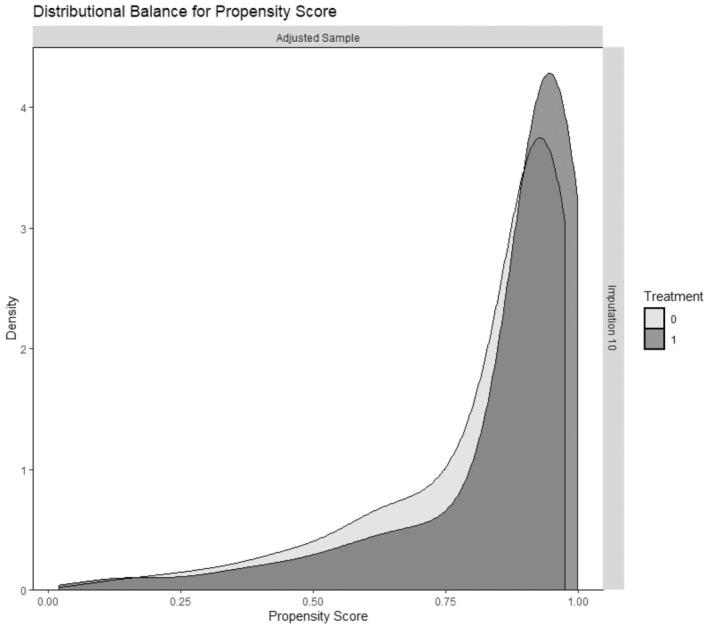
Distributional Balance of the Propensity Score for the Treatment School Type (Exemplary for One Imputed Dataset).

**FIGURE A4:**
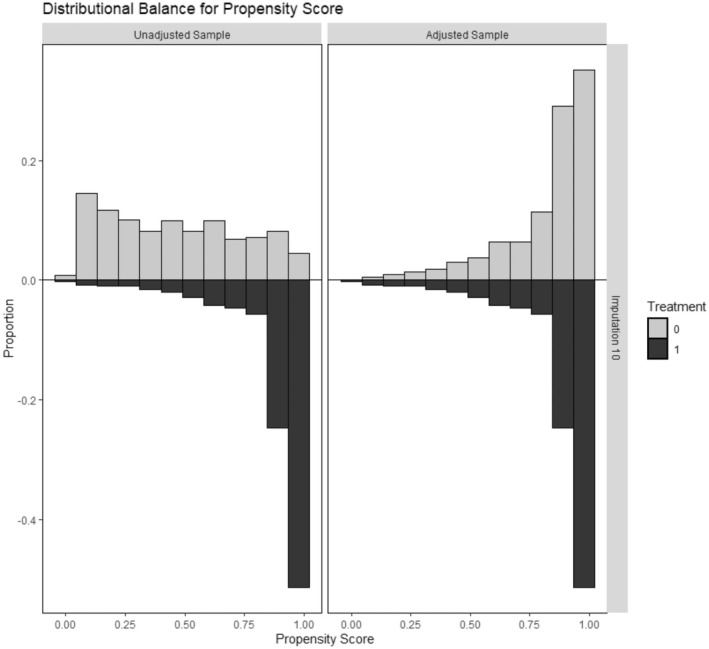
Distributional Balance of the Propensity Score for the Treatment School type (Exemplary for One Imputed Dataset) for the Unadjusted and Adjusted Sample. Treatment: 0 = Non‐academic track (Haupt/Realschule), 1 = Academic track (Gymnasium).

The sample and the quality of the calculated PSs were checked in advance (please see Figures A3 and A4 in the Appendix A). The covariate balance was assessed using the R package *cobalt* (Greifer, 2022). The IPT weights were not stabilized in this analysis. The distribution of the weights across imputations indicates that a few individuals have high weights (Max: 48.661; see also Table A2). However, diagnostic checks showed no convergence issues, good propensity score overlap, successful covariate balance and stable standard errors. These findings suggest that the estimates are robust despite the presence of high weights. Figure 1 shows the standardized mean differences of all covariates among students from academic and non‐academic track schools (for absolute standardized mean differences, please see Figure A2 in the Appendix A). The grey dots represent the mean differences in the unadjusted sample before weighting, while the black dots represent the mean differences in the adjusted sample after weighting. The dotted lines represent the 0.1 threshold, though the literature suggests varying thresholds between 0.1 and 0.25 (Rubin, 2001). The plot demonstrates that most points are within the threshold after weighting, suggesting overall balance has been achieved (Greifer, 2022). The results and additional information are in Tables A1 and A2 in the Appendix A.

**FIGURE 2:**
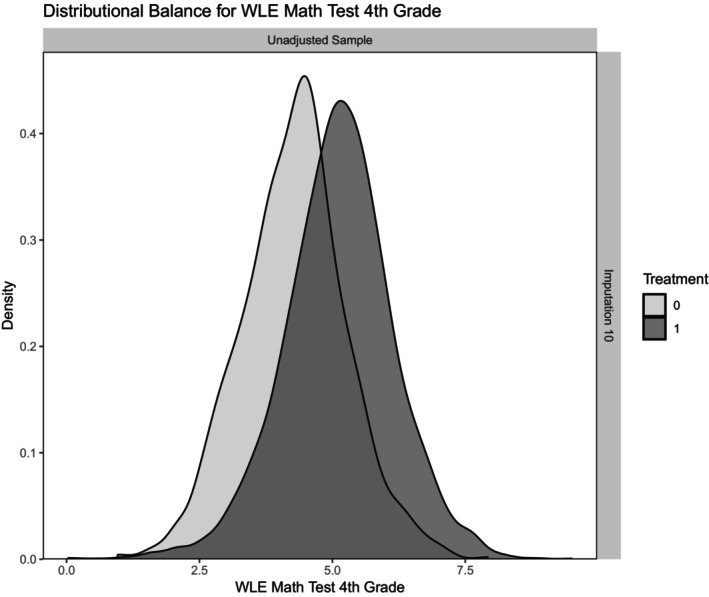
Distributional Balance of the Variable ‘WLE Math Test 4th Grade’ for the Treatment School Type (exemplary for one imputed dataset). Treatment: 0 = Non‐academic track (Haupt/Realschule), 1 = Academic track (Gymnasium).

Figure 2 illustrates the distribution of the PSs of the variable ‘Math Competence Test in Fourth Grade (WLE)’ for both school types, exemplary for one multiple imputed dataset. The graphical comparison of the distributions gives a first impression of their width of overlap and the size of the area of common support—that is, the area in which comparable primary school students can be found. Notably, students who subsequently transferred to an academic track school performed significantly better on the math competence test in the fourth grade. The PSs are approximately normally distributed in both school types, and the distributions overlap in large parts. For more detailed model diagnostics of sample balance and the balance of the propensity score, please see Figures A2 and A3 and Table A1 in the Appendix A.

On average, in each imputed data set, 982 students in the control group and 3198 in the treatment group were used for the analyses. The propensity score analysis (see Table 2) revealed a significant average treatment effect on the treated (ATT) of 0.272. Given the distribution of the WLE (see also Table 1 and Figure A1), this effect can be categorized as moderate. The ATT measures the causal effect of a treatment on those who received the treatment. The ATT indicates that attending an academic track school significantly improves math competence by 0.272 units for the students who attended these schools, compared to if they had attended non‐academic track schools.

**TABLE 2:** Average Treatment Effect on the Treated (ATT) with the Dependent Variable Math Competency 7th grade and Treatment ‘Academic School Track Attendance (Gymnasium)’, *N* = 4180.

ATT	Standardized ATT	Std.Error	CI 95%	*Df*
0.272**	0.229**	0.102	[0.071, 0.474]	117

*p < .05.

** *p* < .01.

***p < .001.

The results suggest that comparable students would have performed better on an academic track school. However, the question arises about whether these effects are pronounced differently for different socio‐economic backgrounds. To answer this question, we conducted an additional propensity score weighting analysis for students with high and low SES. For this purpose, we divided the ISEI‐88 of the anchor parent at the median into two groups (the median is 52). The results in Tables 3 and 4 suggest that the average treatment effect on the treated is more pronounced for students with low SES (ATT = 0.269, *p* < .01), meaning they lag further behind comparable students in the academic track. The effect for children with high SES is 0.291 but not significant (*p* > .05). Please note that the lack of significance in the high SES group may be due to greater heterogeneity in this group and does not necessarily imply the absence of an effect. This heterogeneity can mask clear effects, making them more challenging to identify compared to more homogeneous groups.

**TABLE 3:** Average Treatment Effect on the Treated (ATT) with the dependent variable math competency 7th grade and treatment ‘Academic School Track Attendance (Gymnasium)’ for students with low SES, *N* = 2130.

ATT	Standardized ATT	Std.Error	CI 95%	*Df*
0.269**	0.226**	0.108	[0.056, 0.481]	165

* *p* < .05.

** *p* < .01.

*** *p* < .001.

**TABLE 4:** Average Treatment Effect on the Treated (ATT) with the Dependent Variable Math Competency 7th Grade and Treatment ‘Academic School Track Attendance (Gymnasium)’ for Students with High SES, *N* = 2050.

ATT	Standardized ATT	Std.Error	CI 95%	*Df*
0.291	0.245	0.148	[−0.002, 0.583]	131

Additional statistical testing between both ATTs indicates that the difference between these effects is not significant (*p* > .05), suggesting that the influence of attending a non‐academic track on Math competencies is similarly large for both groups (Table 5). Overall, there is no evidence that the effect of attending a non‐academic track differs substantially between SES groups. Attending a non‐academic track likely has comparably detrimental effects for both groups.

**TABLE 5:** Test of statistical significance of the difference between the ATTs of students with high and low SES.

ATT_SESlow_	ATT_SEShigh_	N_SESlow_	N_SEShigh_	SE_SESlow_	SE_SEShigh_	ATT diff	SE diff	*Z*	*p*
0.291	0.272	2130	2050	0.148	0.108	0.019	0.183	0.104	.917

## DISCUSSION

Our study highlights a crucial disparity: Children with comparable math competencies in the fourth grade perform significantly worse after transferring to a non‐academic track school than if they had attended an academic track school. Our quasi‐experimental analysis suggests that the school types and learning environments have a causal effect on students' competence development. The results align with research applying similar methods conducted in Germany, such as the recent study by Traini et al. (2021), which also found clear performance differences between students at different tracks despite similar preconditions. While Traini et al. (2021) used entropy balancing to account for pre‐existing differences, their data was collected after tracking had already taken place, limiting their ability to draw causal conclusions. In contrast, our study employs a quasi‐experimental design with data collected before tracking, allowing us to make stronger causal inferences.

Low SES and disadvantageous home learning environments are often considered as one of the primary factors for poor performance (Croizet et al., 2001; Hofer et al., 2023). Our study focuses not on individual characteristics but emphasizes the institutional disadvantage of attending a non‐academic track school regardless of students' demographic characteristics. By comparing students who are similar in terms of their SES, abilities, aspirations and other characteristics but attend different school types, we found that the latter seems most influential on their math competence development, as indicated by their performance. A closer look at SES differences reveals that the treatment effect is more pronounced for students with low SES in non‐academic tracks, indicating that they are at a greater disadvantage compared to their peers in academic tracks, which might be a result of, e.g., the lower level of academic support available at home. As such, tracking largely accounts for the educational gap based on SES (Dräger et al., 2024). Additionally, the greater heterogeneity within the high SES group should be considered, as this diversity tends to dilute the results for this group, potentially masking clearer patterns or trends. However, we found no evidence of a substantial difference in the track effect across SES groups overall. The adverse effects of attending a non‐academic track appear similarly disadvantageous for both groups. This aligns with findings suggesting that non‐academic tracks can negatively influence academic outcomes and future opportunities, regardless of a student's SES background (Biedermann et al., 2015; Hanushek & Wößmann, 2006).

Our findings suggest that students with math competences comparable to those in academic tracks may not fully realize their potential in non‐academic track schools. These barriers to competence development have long‐term consequences for students' educational, personal, and general labor market outcomes (Ozer & Perc, 2020). First, transitioning to a higher track later on is rare and challenging. Only approximately 15% of students change between school tracks in general (Cortina, 2003; Mauthe & Rösner, 1998), and students in non‐academic tracks mostly remain in non‐academic tracks (Blossfeld, 2018). Therefore, the current system of tracking, heavily influenced by teacher recommendations and parental decisions (Batruch et al., 2019; Lüdemann & Schwerdt, 2013), poses significant risks, particularly for marginalized groups, including students from low SES or migrant backgrounds. Furthermore, placement in a non‐academic track can negatively impact students' self‐concept, leading to insecurity about their academic and general futures (Ireson et al., 2001; Oakes, 2005). International examples, such as France's detracking reforms, show that addressing these issues can improve educational outcomes and economic opportunities, especially for lower SES students (Canaan, 2020).

Second, the economic and societal implications are notable. The inefficient use of potential in non‐academic track students, particularly in the context of a skilled labor shortage, represents a missed opportunity for the labor market (Romer, 1990).

While our study clearly shows the impact of school types on math competence development, it does not uncover the specific school‐related factors driving these differences. However, Traini et al. (2021) partially address this gap by showing that class performance composition and prior skills mediate these effects. Their findings suggest that the compositional effects, outlined in the framework by Baumert et al. (2006), contribute to performance differences between academic‐ and non‐academic track schools. Nevertheless, further research is needed to fully understand how these mechanisms function and the specific factors that foster or hinder competence development across different tracks.

A particular strength of our study lies in its broad range of matching variables compared to, e.g., Traini et al. (2021). While they focused on socioeconomic factors and general student characteristics, we were able to include essential variables such as students' aspirations, grades from the fourth grade, and teachers' school recommendations. This allows us to isolate the effects of school type more precisely and better account for the influence of individual expectations and prior academic performance.

### Implications for practice

In practical terms, the results underscore the need for alternatives in secondary school recommendations to avoid misallocation. Current practices rely mainly on subjective teacher assessment and parental choice. Generally, considering the long‐term adverse effects of underestimating (rather than overestimating) students’ abilities, especially for marginalized students, teachers could give students the benefit of the doubt when their performance is borderline. Introducing objective measures, such as entrance qualification tests or external evaluations, could enhance the fairness of school placement decisions, particularly for students from lower SES or migrant backgrounds. Such measures could also mitigate the compositional effects that Traini et al. (2021) identified, ensuring a more equitable selection process.

Furthermore, it would be beneficial to facilitate transitions to other school tracks later on through programs that allow students to qualify for the higher track. Additionally, an official protocol for early detection of misattribution within the first six months of tracking might help prevent potential delays in academic learning for students who were misattributed.

In addition, addressing the compositional effects of tracking systems is essential. While tracking persists, non‐academic track students are often placed in less favourable learning environments. To counteract this, schools should implement targeted interventions, such as one‐on‐one tutoring and mentoring programs, which have been shown to boost motivation and academic performance (Lee & Cramond, 1999). Moreover, increasing the presence of social workers and counsellors can help meet the needs of students from challenging backgrounds, fostering a more conducive learning environment and reducing disruptive behaviours (Early & Vonk, 2001).

Finally, improving teaching quality in non‐academic track schools is crucial. Aligning teacher training across tracks and focusing on fostering student potential can help bridge the performance gap between school types. Such efforts are critical to achieving greater educational equity (Ditton et al., 2005; Solga & Wagner, 2001).

### Limitations and further research

While our research design provides insights into how attending a non‐academic track compared to an academic track school impacts math competence for comparable students, it was not possible to determine the reasons behind the differences in competence development. Determining precise causal factors that negatively affect the competence development of students eligible for an academic track school but attending a non‐academic track school and the associated mechanisms remain to be investigated.

Moreover, it is important to mention that students' aptitude is measured mainly in terms of math and reading competences, school grades and other important characteristics such as SES, gender and student body of the elementary school. Of course, other factors, such as maturity or other private reasons, can play a fundamental role in choosing an academic track school. Unfortunately, these factors have remained unconsidered since no information is available in the data.

Our focus on math competence was influenced by the available data and prior research emphasizing the strong impact of ability grouping on Math (e.g., Ireson, Clark, & Hallam, 2002). Future studies could expand this focus to other subjects and explore psychological and emotional characteristics that might be affected by track placement.

The issue of misallocation is not exclusive to non‐academic tracks. Incorrect placement in academic tracks can also lead to adverse outcomes such as dropouts, diminished self‐concept (e.g., big‐fish‐little‐pond effect; Marsh, 1987) and limited career opportunities (Köller & Baumert, 2001; Pfost et al., 2018). Further investigation is needed to understand the impact of misallocation in both directions, considering individual and affective traits.

Our study can directly attribute competence stagnation in non‐academic track schools to the influence of the school type. This is an advantage compared to Traini et al. (2021), who mainly focused on compositional and social background effects between school tracks but could not determine whether the tracking caused these differences or whether the students would have performed worse regardless of the track. Nevertheless, our findings invite further inquiry about which specific institutional and structural characteristics limit competence development, such as subpar teaching quality or unfavourable learning environments in non‐academic track schools (e.g., Esser & Relikowski, 2015; Guill et al., 2017). Additionally, composition effects and the influence of a less motivated student body could have a decisive impact on competence development depending on the school type (Biedermann et al., 2015; Hanushek & Wößmann, 2006; Hattie, 2002). Future research could shed light on how these institutional and structural factors interact within non‐academic track schools to impact student competence development.

Moreover, panel studies like NEPS are subject to attrition, particularly after the transition to secondary school, which may introduce bias. Furthermore, the overrepresentation of higher SES students in the NEPS data (Zinn et al., 2020) could underestimate the effects on lower SES students. Additionally, math assessments were conducted at long intervals, limiting our ability to track competence development more precisely. As NEPS continues, future research could take advantage of additional data points to examine competency development more closely throughout secondary education.

Finally, the long‐term educational and career‐related outcomes of misallocated students should be explored further. Future research could investigate whether misclassified students are more likely to pursue university entrance through vocational training programs and examine any potential long‐term psychological or career‐related disadvantages.

## CONCLUSION

Previous research has often attributed competence differences to socioeconomic factors such as parental education and home learning environments (e.g., Alexander et al., 2001; Crawford et al., 2017; Ditton & Krüsken, 2009). However, a direct comparison of similarly capable students at the end of elementary school, attending different secondary school types, had not been conducted in Germany until now. Our study fills this gap, showing that students with initially similar competencies and backgrounds diverge significantly in their competency development depending on whether they attend academic‐ or non‐academic track schools.

Our PSW analyses reveal that students at non‐academic track schools fall behind their peers at academic track schools, regardless of their initial Math abilities or high SES backgrounds. This suggests that neither individual academic potential nor parental support can fully counterbalance the institutional and compositional disadvantages of non‐academic track schools. These findings go beyond those of Traini et al. (2021), as we demonstrate that school type, rather than pre‐existing socioeconomic factors, plays a critical role in shaping students' competence development. Traini et al. (2021) used entropy balancing to adjust for pre‐existing differences, but their post‐tracking data limited causal conclusions. In contrast, our quasi‐experimental design enables stronger causal inferences.

Furthermore, there is no indication that the impact of enrolling in a non‐academic track varies significantly among different socio‐economic status (SES) groups. The adverse effects of attending a non‐academic track appear similarly detrimental for both groups.

Moreover, the consequences of a student's misplacement can be extensive, as transferring to an academic‐track school is challenging. Students' impaired performance can also lead to psychological repercussions, potentially damaging their self‐concept and self‐esteem (e.g., Ireson et al., 2001), which can, in turn, further limit academic performance (Lauermann et al., 2020; Marsh & Martin, 2011).

Our findings underline the importance of addressing misallocations in the tracking system. We recommend developing a more objective and transparent process for school recommendations, reducing reliance on subjective factors like teacher judgement or parental influence. Additionally, our results challenge the assumption that SES is the primary determinant of academic success. Instead, the type of school plays a crucial and underappreciated role in shaping students' academic trajectories. This insight offers a new perspective on educational equity, highlighting the need to reform school placement procedures to ensure all students reach their full potential.

## AUTHOR CONTRIBUTIONS

**Sonja Herrmann:** Conceptualization; writing – original draft; writing – review and editing; supervision; formal analysis; methodology; data curation; investigation. **Katharina M. Bach:** Writing – original draft; writing – review and editing.

## CONFLICT OF INTEREST STATEMENT

All authors declare no conflict of financial or non‐financial interest.

## Data Availability

The National Educational Panel Study (NEPS): Starting Cohort 2—Kindergarten, doi:10.5157/NEPS:SC2:1.0.0. From 2008 to 2013 date are freely available to the scientific community.

## Footnotes

^1^
Note that there are few federal states (Berlin and Brandenburg) where the tracking occurs after the sixth grade, approximately at the age of 12.

^2^
In the federal states of Bavaria, Thuringia and Brandenburg, the secondary school recommendation is binding. However, in these three federal states, parents can still choose a track that is lower than the recommended one. For an overview of differences among the 16 German federal states, see Helbig & Nikolai, 2015; Henninges, et al., 2019.

^3^
For detailed information on the sampling procedure, see Hellrung et al. (2011) and Steinhauer et al. (2016).

^4^
For readers interested in a more detailed description of all competence tests, we recommend the detailed documentation on competence tests at https://www.neps‐data.de/Data‐Center/Data‐and‐Documentation/Start‐Cohort‐Kindergarten/Documentation.

## APPENDIX A

### A.1

**TABLE A1:** Balance summary across all imputations.

	Min.Diff.	Mean.Diff.	Max.Diff.
WLE Math Test 4th grade	0.83	0.84	0.87
WLE Reading Test 4th grade	0.32	0.35	0.38
Grade Math	−0.54	−0.51	−0.48
Grade German	−0.62	−0.60	−0.57
Special educational needs (Ref. no)	−0.04	−0.02	0.12
Former GDR (Ref. West Germany)	−0.09	−0.03	−0.03
Enrolment in primary school—regular	–	–	–
*Enrolment in primary school*—*early*	0.04	0.19	0.24
*Enrolment in primary school*—*late*	−0.24	−0.16	−0.08
Idealistic aspirations (Ref. (Hauptschule))	–	–	–
*Upper non‐academic track (Realschule)*	−0.87	−0.84	−0.81
*Academic track (Abitur)*	0.92	0.95	0.97
Realistic aspirations (Ref. (Hauptschule))		–	–
*Upper non‐academic track (Realschule)*	−0.90	−0.85	−0.82
*Academic track (Abitur)*	0.97	1.03	1.08
Anticipated secondary school recommendation	1.22	1.29	1.36
Change of secondary school type	−0.09	−0.09	−0.09
Sex (Ref. male)	0.01	−0.03	0.05
Migration background (Ref. none)	−0.08	−0.05	−0.02
SES anchor ISEI‐88	0.46	0.47	0.49
SES partner ISEI‐88	0.53	0.58	0.62
Class: Amount of Math lessons	−0.03	−0.00	0.03
Class: Share of higher educated parents	0.21	0.26	0.32
Class: Share females	−0.12	−0.08	−0.04
Average sample sizes across all imputations	Control		Treated
	982		3198

**TABLE A2:** Summary statistics for weights across imputations.

Min	Median	Mean	Max
0.0234	1.0	1.372	48.661

## References

[bjep12741-bib-0001] Alexander, K. L. , Entwisle, D. R. , & Olson, L. S. (2001). Schools, achievement, and inequality: A seasonal perspective. Educational Evaluation and Policy Analysis, 23(2), 171–191. 10.3102/01623737023002171

[bjep12741-bib-0002] Angelone, D. (2019). Schereneffekte auf der Sekundarstufe I? Zum Einfluss des Schultyps auf den Leistungszuwachs in Deutsch und Mathematik. Swiss Journal of Educational Research, 41(2), 446–466. 10.24452/sjer.41.2.11

[bjep12741-bib-0003] Arbeitsgruppe Bildungsbericht am Max‐Planck‐Institut für Bildungsforschung . (1994). Das bildungssystem in der bundesrepublik deutschland: strukturen und entwicklungen im überblick [The education system in the Federal Republic of Germany: An overview of structures and developments]. Rowohlt.

[bjep12741-bib-0004] Argys, L. M. , Rees, D. I. , & Brewer, D. J. (1996). Detracking America's schools: Equity at zero cost? Journal of Policy Analysis and Management, 15(4), 623–645. 10.1002/(SICI)1520-6688(199623)15:4<623::AID-PAM7>3.0.CO;2-J

[bjep12741-bib-0005] Ariga, K. , & Brunello, G. (2007). Does secondary school tracking affect performance? Evidence from IALS (No. 2643; IZA Discussion Papers). Institute for the Study of Labor (IZA). https://papers.ssrn.com/abstract=970246

[bjep12741-bib-0006] Austin, P. C. (2011). An introduction to propensity score methods for reducing the effects of confounding in observational studies. Multivariate Behavioral Research, 46(3), 399–424. 10.1080/00273171.2011.568786 21818162 PMC3144483

[bjep12741-bib-0007] Banerjee, P. A. (2016). A systematic review of factors linked to poor academic performance of disadvantaged students in science and maths in schools. Cogent Education, 3(1), 1178441.

[bjep12741-bib-0008] Barth, D. , Jessen, J. , Spieß, C. K. , & Wrohlich, K. (2020). Mütter in Ost und West: Angleichung bei erwerbstätigenquoten und einstellungen, nicht bei vollzeiterwerbstätigkeit [Mothers in East and West: Equalization in employment rates and hiring, not in full‐time employment]. DIW Wochenbericht, 87(38), 699–706. 10.18723/diw_wb:2020-38-2

[bjep12741-bib-0009] Batruch, A. , Autin, F. , Bataillard, F. , & Butera, F. (2019). School selection and the social class divide: How tracking contributes to the reproduction of inequalities. Personality and Social Psychology Bulletin, 45(3), 477–490. 10.1177/0146167218791804 30111241

[bjep12741-bib-0010] Baumert, J. , Becker, M. , Neumann, M. , & Nikolova, R. (2010). Besondere Förderung von Kernkompetenzen an Spezialgymnasien? [special promotion of core competencies at special grammar schools?]. Zeitschrift für Pädagogische Psychologie, 24(1), 5–22. 10.1024/1010-0652/a000001

[bjep12741-bib-0011] Baumert, J. , Klieme, E. , Neubrand, M. , Prenzel, M. , Schiefeke, U. , Schneider, W. , Stanat, P. , Tillmann, K.‐J. , & Weiß, M. (2001). PISA 2000: Basiskompetenzen von Schülerinnen und Schülern im internationalen Vergleich [PISA 2000: An international comparison of students' basic skills]. Leske + Budrich.

[bjep12741-bib-0012] Baumert, J. , & Köller, O. (2005). Sozialer Hintergrund, Bildungsbeteiligung und Bildungsverläufe im differenzierten Sekundarschulsystem [social background, educational participation and educational trajectories in the differentiated secondary school system]. In V. Frederking , H. Heller , & A. Scheunpflug (Eds.), Nach PISA (pp. 9–21). VS Verlag für Sozialwissenschaften. 10.1007/978-3-322-80658-1_2

[bjep12741-bib-0013] Baumert, J. , Stanat, P. , & Watermann, R. (2006). Schulstruktur und die Entstehung differenzieller Lern‐und Entwicklungsmilieus [School structure and the emergence of differentiated learning and development environments] (pp. 95–188). Herkunftsbedingte Disparitäten im Bildungswesen: Differenzielle Bildungsprozesse und Probleme der Verteilungsgerechtigkeit: Vertiefende Analysen im Rahmen von PISA 2000. 10.1007/978-3-531-90082-7_4

[bjep12741-bib-0014] Becker, M. , Kocaj, A. , Jansen, M. , Dumont, H. , & Lüdtke, O. (2022). Class‐average achievement and individual achievement development: Testing achievement composition and peer spillover effects using five German longitudinal studies. Journal of Educational Psychology, 114(1), 177–197. 10.1037/edu0000519

[bjep12741-bib-0015] Becker, M. , Lüdtke, O. , Trautwein, U. , Köller, O. , & Baumert, J. (2012). The differential effects of school tracking on psychometric intelligence: Do academic track schools make students smarter? Journal of Educational Psychology, 104(3), 682–699. 10.1037/a0027608

[bjep12741-bib-0016] Becker, M. , McElvany, N. , Lüdtke, O. , & Trautwein, U. (2014). Lesekompetenzen und schulische Lernumwelten. [Reading skills and school learning environments]. Zeitschrift für Entwicklungspsychologie Und Pädagogische Psychologie, 46(1), 35–50. 10.1026/0049-8637/a000104

[bjep12741-bib-0017] Biedermann, H. , Weber, C. , Herzog‐Punzenberger, B. , & Nagel, A. (2015). Auf die Mitschüler/innen kommt es an? Schulische Segregation—Effekte der Schul‐und Klassenzusammensetzung in der Primarstufe und der Sekundarstufe I [Does it depend on the classmates? School segregation‐effects of school and class composition at primary and lower secondary level]. Nationaler Bildungsbericht Österreich, 2, 133–174.

[bjep12741-bib-0018] Bloom, B. S. (1976). Human characteristics and school learning. McGraw‐Hill.

[bjep12741-bib-0019] Blossfeld, H. , Roßbach, H. , & von Maurice, J. (2011). The German national educational panel study (NEPS). Zeitschrift für Erziehungswissenschaft, Sonderheft, 14, 215–230. 10.1007/978-3-658-23162-0

[bjep12741-bib-0020] Blossfeld, P. (2018). Social background and between‐track mobility in the general education system in West Germany and in East Germany after German unification. Zeitschrift für Soziologie, 47(4), 255–269. 10.1515/zfsoz-2018-0117

[bjep12741-bib-0021] Bonefeld, M. , & Dickhäuser, O. (2018). (Biased) grading of students' performance: Students' names, performance level, and implicit attitudes. Frontiers in Psychology, 9, 481. 10.3389/fpsyg.2018.00481 29867618 PMC5954233

[bjep12741-bib-0022] van Buuren, S. , & Groothuis‐Oudshoorn, K. (2011). Mice: Multivariate imputation by chained equations in R. Journal of Statistical Software, 45, 1–67. 10.18637/jss.v045.i03

[bjep12741-bib-0023] Canaan, S. (2020). The long‐run effects of reducing early school tracking. Journal of Public Economics, 187, 104206.

[bjep12741-bib-0024] Card, D. , & Giuliano, L. (2016). Can tracking raise the test scores of high‐ability minority students? American Economic Review, 106(10), 2783–2816. 10.1257/aer.20150484

[bjep12741-bib-0025] Condron, D. J. (2008). An early start: Skill grouping and unequal reading gains in the elementary years. The Sociological Quarterly, 49(2), 363–394. 10.1111/j.1533-8525.2008.00119.x

[bjep12741-bib-0026] Connolly, P. , Taylor, B. , Francis, B. , Archer, L. , Hodgen, J. , Mazenod, A. , & Tereshchenko, A. (2019). The misallocation of students to academic sets in maths: A study of secondary schools in England. British Educational Research Journal, 45(4), 873–897. 10.1002/berj.3530

[bjep12741-bib-0027] Cortina, K. , Baumert, J. , Leschinsky, A. , Mayer, K. , & Trommer, L. (2008). Das Bildungswesen in der Bundesrepublik Deutschland. In Strukturen und Entwicklungen im Überblick [education in the Federal Republic of Germany. Structures and developments at a glance] (3rd ed.). Rowohlt Taschenbuch Verlag.

[bjep12741-bib-0028] Cortina, K. S. (2003). Der Schulartwechsel in der Sekundarstufe I: pädagogische Maßnahme oder Indikator eines falschen Systems? [Changing school type at lower secondary level: pedagogical measure or indicator of a wrong system?]. Zeitschrift für Pädagogik, 49(1), 127–141.

[bjep12741-bib-0029] Crawford, C. , Macmillan, L. , & Vignoles, A. (2017). When and why do initially high‐achieving poor children fall behind? Oxford Review of Education, 43(1), 88–108. 10.1080/03054985.2016.1240672

[bjep12741-bib-0117] Croizet, J. C. , Désert, M. , Dutrévis, M. , & Leyens, J.‐P. (2001). Stereotype threat, social class, gender, and academic under‐achievement: when our reputation catches up to us and takes over. Social Psychology of Education, 4, 295–310. 10.1023/A:1011336821053

[bjep12741-bib-0030] Ditton, H. , & Krüsken, J. (2006). Der Übergang von der Grundschule in die Sekundarstufe I The transition from elementary school to lower secondary level. Zeitschrift für Erziehungswissenschaft, 9(3), 348–372. 10.1007/s11618-006-0055-7

[bjep12741-bib-0031] Ditton, H. , & Krüsken, J. (2009). Denn wer hat, dem wird gegeben werden? Eine Längsschnittstudie zur Entwicklung schulischer Leistungen und den Effekten der sozialen Herkunft in der Grundschulzeit [For he who has, to him shall be given? A longitudinal study on the development of academic performance and the effects of social background in primary school]. Journal for Educational Research Online, 1(1), 33–61.

[bjep12741-bib-0032] Ditton, H. , Krüsken, J. , & Schauenberg, M. (2005). Bildungsungleichheit—Der Beitrag von Familie und Schule [educational inequality‐the contribution of family and school]. Zeitschrift für Erziehungswissenschaft, 8(2), 285–304.

[bjep12741-bib-0033] Domina, T. , Penner, A. , & Penner, E. (2017). Categorical inequality: Schools as sorting machines. Annual Review of Sociology, 43, 311–330. 10.1146/annurev-soc-060116-053354 PMC5892435 29657353

[bjep12741-bib-0034] Dräger, J. , Schneider, T. , Olczyk, M. , Solaz, A. , Sheridan, A. , Washbrook, E. , Perinetti Casoni, V. , Kwon, S. J. , & Waldfogel, J. (2024). The relevance of tracking and social school composition for growing achievement gaps by parental education in lower secondary school: A longitudinal analysis in France, Germany, the United States, and England. European Sociological Review, 40(6), 964–980. 10.1093/esr/jcad076

[bjep12741-bib-0035] Dumont, H. , Neumann, M. , Maaz, K. , & Trautwein, U. (2013). Die Zusammensetzung der Schülerschaft als Einflussfaktor für Schulleistungen. Psychologie in Erziehung und Unterricht, 60(3), 163–183. 10.2378/peu2013.art14d

[bjep12741-bib-0036] Dustmann, C. (2004). Parental background, secondary school track choice, and wages. Oxford Economic Papers, 56(2), 209–230.

[bjep12741-bib-0037] Early, T. J. , & Vonk, M. E. (2001). Effectiveness of school social work from a risk and resilience perspective. Children & Schools, 23(1), 9–31.

[bjep12741-bib-0038] Esser, H. , & Relikowski, I. (2015). Is ability tracking (really) responsible for educational inequalities in achievement? A comparison between the country states Bavaria and Hesse in Germany (Vol. 9082, p. 34). IZA Discussion Papers, Institute for the Study of Labor (IZA).

[bjep12741-bib-0039] Faust, G. (2006). Zum Stand der Einschulung und der neuen Schuleingangsstufe in Deutschland. Zeitschrift für Erziehungswissenschaft, 9(3), 328–347. 10.1007/s11618-006-0054-8

[bjep12741-bib-0040] Fischer, L. , Rohm, T. , Gnambs, T. , & Carstensen, C. H. (2016). Linking the data of the competence tests (NEPS Survey Paper No. 1). Leibniz Institute for Educational Trajectories, National Educational Panel Study. 10.5157/NEPS:SP01:1.0

[bjep12741-bib-0041] Frey, A. , Heinze, A. , Mildner, D. , Hochweber, J. , & Asseburg, R. (2010). Mathematische Kompetenz von PISA 2003 bis PISA 2009 [Mathematical competence from PISA 2003 to PISA 2009]. In E. Klieme , C. Artelt , J. Hartig , N. Jude , O. Köller , M. Prenzel , W. Schneider , & P. Stanat (Eds.), PISA 2009: Bilanz nach einem Jahrzehnt (pp. 153–176). Waxmann.

[bjep12741-bib-0042] Gamoran, A. , & Mare, R. D. (1989). Secondary school tracking and educational inequality: Compensation, reinforcement, or neutrality? American Journal of Sociology, 94(5), 1146–1183. 10.1086/229114

[bjep12741-bib-0043] Ganzeboom, H. B. G. , De Graaf, P. M. , & Treiman, D. J. (1992). A standard international socio‐economic index of occupational status. Social Science Research, 21(1), 1–56. 10.1016/0049-089X(92)90017-B

[bjep12741-bib-0044] Göllner, R. , Damian, R. I. , Nagengast, B. , Roberts, B. W. , & Trautwein, U. (2018). It's not only who you are but who you are with: High school composition and individuals' attainment over the life course. Psychological Science, 29(11), 1785–1796. 10.1177/0956797618794454 30215575

[bjep12741-bib-0045] Greifer, N. (2022). *cobalt: covariate balance tables and plots*. (R package version 4.4.0.). https://cran.r‐project.org/web/packages/cobalt/vignettes/cobalt.html

[bjep12741-bib-0046] Guill, K. , Lüdtke, O. , & Köller, O. (2017). Academic tracking is related to gains in students' intelligence over four years: Evidence from a propensity score matching study. Learning and Instruction, 47, 43–52. 10.1016/j.learninstruc.2016.10.001

[bjep12741-bib-0047] Gujare, S. K. , & Tiwari, G. K. (2016). Academic self‐concept and academic outcome of the graduate students: The mediating role of socioeconomic status and gender. International Journal of Education and Psychological Research, 4(5), 1–7.

[bjep12741-bib-0048] Haeberlin, U. , Imdorf, C. , & Kronig, W. (2004). Chancenungleichheit bei der Lehrstellensuche: Der Einfluss von Schule, Herkunft und Geschlecht [Unequal opportunities when looking for an apprenticeship: The influence of school, origin and gender]. Schweizerischer Nationalfonds, Nationales Forschungsprogramm «Bildung und Beschäftigung» (NFPNR 43).

[bjep12741-bib-0049] Hanushek, E. A. , & Wößmann, L. (2006). Does educational tracking affect performance and inequality? Differences‐in‐differences evidence across countries. The Economic Journal, 116(510), C63–C76. 10.1111/j.1468-0297.2006.01076.x

[bjep12741-bib-0050] Hattie, J. A. C. (2002). Classroom composition and peer effects. International Journal of Educational Research, 37(5), 449–481. 10.1016/S0883-0355(03)00015-6

[bjep12741-bib-0051] Helbig, M. , & Nikolai, R. (2015). Die Unvergleichbaren: der Wandel der Schulsysteme in den deutschen Bundesländern seit 1949 [The incomparable: the transformation of school systems in the German federal states since 1949]. Julius Klinkhardt.

[bjep12741-bib-0116] Hellrung, M. , Waschk, A. , Oberlein, J. , & Hillen, P. (2011). Methodenbericht: NEPS Startkohorte 2 Haupterhebung – Winter/Frühjahr/Sommer 2011 (A12). IEA Data Processing and Research Center.

[bjep12741-bib-0052] Henninges, M. , Traini, C. , & Kleinert, C. (2019). Tracking and sorting in the German educational system. Leibniz Institute for Educational Trajectories (LIfBi) Working Paper No, 83.

[bjep12741-bib-0053] Hofer, S. I. , Reinhold, F. , & Koch, M. (2023). Students home alone—Profiles of internal and external conditions associated with mathematics learning from home. European Journal of Psychology of Education, 38(1), 333–366. 10.1007/s10212-021-00590-w PMC8727485 40477537

[bjep12741-bib-0054] Hoffer, T. B. (1992). Middle school ability grouping and student achievement in science and mathematics. Educational Evaluation and Policy Analysis, 14(3), 205–227. 10.3102/01623737014003205

[bjep12741-bib-0055] Hosenfeld, I. , Köller, O. , & Baumert, J. (1999). Why sex differences in mathematics achievement disappear in German secondary schools: A reanalysis of the German TIMSS‐data. Studies in Educational Evaluation, 25(2), 143–161. 10.1016/S0191-491X(99)00018-8

[bjep12741-bib-0056] Hoyer, R. S. , Pakulak, E. , Bidet‐Caulet, A. , & Karns, C. M. (2023). Relationships among age, socioeconomic status, and distractibility in preschoolers as assessed by the competitive attention test. Journal of Experimental Child Psychology, 227, 105584. 36413871 10.1016/j.jecp.2022.105584

[bjep12741-bib-0057] Hurrelmann, K. (1986). Schulerfolg und Schulversagen im Jugendalter: fallanalyzen von Bildungslaufbahnen. Juventa‐Verlag.

[bjep12741-bib-0058] Ireson, J. , Clark, H. , & Hallam, S. (2002). Constructing ability groups in the secondary school: Issues in practice. School Leadership & Management, 22(2), 163–176.

[bjep12741-bib-0059] Ireson, J. , Hallam, S. , Hack, S. , Clark, H. , & Plewis, I. (2002). Ability grouping in English secondary schools: Effects on attainment in English, mathematics and science. Educational Research and Evaluation, 8(3), 299–318. 10.1076/edre.8.3.299.3854

[bjep12741-bib-0060] Ireson, J. , Hallam, S. , & Plewis, I. (2001). Ability grouping in secondary schools: Effects on pupils' self‐concepts. British Journal of Educational Psychology, 71(2), 315–326. 10.1348/000709901158541 11449939

[bjep12741-bib-0061] Kolenikov, S. , & Bollen, K. A. (2012). Testing negative error variances: Is a Heywood case a symptom of misspecification? Sociological Methods & Research, 41(1), 124–167. 10.1177/0049124112442138

[bjep12741-bib-0062] Köller, O. , & Baumert, J. (2001). Leistungsgruppierungen in der Sekundarstufe I. Ihre Konsequenzen für die Mathematikleistung und das mathematische Selbstkonzept der Begabung [performance groupings in lower secondary school. Their consequences for mathematics performance and the mathematical self‐concept of giftedness]. Zeitschrift Für Pädagogische Psychologie, 15(2), 99–110. 10.1024/1010-0652.15.2.99

[bjep12741-bib-0063] Köller, O. , Schütte, K. , Zimmermann, F. , Retelsdorf, J. , & Leucht, M. (2013). Starke Klasse, hohe Leistungen? [strong class, high performance?] Psychologie in Erziehung und Unterricht, 60(3), 184–197.

[bjep12741-bib-0064] Kristen, C. (2002). Hauptschule, Realschule oder Gymnasium? Ethnische Unterschiede am ersten Bildungsübergang [Hauptschule, Realschule or Gymnasium? Ethnic differences at the first educational transition]. Kölner Zeitschrift für Soziologie Und Sozialpsychologie, 54(3), 534–552.

[bjep12741-bib-0065] Kunter, M. , Brunner, M. , Baumert, J. , Klusmann, U. , Krauss, S. , Blum, W. , Jordan, A. , & Neubrand, M. (2005). Der Mathematikunterricht der PISA‐Schülerinnen und ‐Schüler: Schulformunterschiede in der Unterrichtsqualität Mathematics teaching among PISA students: differences in the quality of teaching between school types. Zeitschrift für Erziehungswissenschaft, 8(4), 502–520. 10.1007/s11618-005-0156-8

[bjep12741-bib-0066] Lauermann, F. , Meißner, A. , & Steinmayr, R. (2020). Relative importance of intelligence and ability self‐concept in predicting test performance and school grades in the math and language arts domains. Journal of Educational Psychology, 112(2), 364–383. 10.1037/edu0000377

[bjep12741-bib-0067] Lauterbach, W. , & Fend, H. (2016). Educational mobility and equal opportunity in different German tracking systems: Findings from the LifE study (pp. 93–109). Models of secondary education and social inequality. An international comparison. 10.4337/9781785367267.00015

[bjep12741-bib-0068] Lee, J. , & Cramond, B. (1999). The positive effects of mentoring economically disadvantaged students. Professional School Counseling, 2(3), 172.

[bjep12741-bib-0069] Lee, J. H. , & Huber, J. C. (2021). Evaluation of multiple imputation with large proportions of missing data: How much is too much? Iranian Journal of Public Health, 50(7), 1372–1380. 10.18502/ijph.v50i7.6626 34568175 PMC8426774

[bjep12741-bib-0070] van Leest, A. , Hornstra, L. , van Tartwijk, J. , & van de Pol, J. (2021). Test‐or judgement‐based school track recommendations: Equal opportunities for students with different socio‐economic backgrounds? British Journal of Educational Psychology, 91(1), 193–216. 10.1111/bjep.12356 32458427 PMC7984160

[bjep12741-bib-0071] Leyrat, C. , Seaman, S. R. , White, I. R. , Douglas, I. , Smeeth, L. , Kim, J. , Resche‐Rigon, M. , Carpenter, J. R. , & Williamson, E. J. (2019). Propensity score analysis with partially observed covariates: How should multiple imputation be used? Statistical Methods in Medical Research, 28(1), 3–19. 10.1177/0962280217713032 28573919 PMC6313366

[bjep12741-bib-0072] Lüdemann, E. , & Schwerdt, G. (2013). Migration background and educational tracking. Journal of Population Economics, 26(2), 455–481.

[bjep12741-bib-0073] Maaz, K. , Neumann, M. , Trautwein, U. , Wendt, W. , Lehmann, R. , & Baumert, J. (2008). Der Übergang von der Grundschule in die weiterführende Schule: die Rolle von Schüler‐ und Klassenmerkmalen beim Einschätzen der individuellen Lernkompetenz durch die Lehrkräfte. [The transition from primary to secondary school: The role of student and class characteristics in teachers' assessment of individual learning competence]. Swiss Journal of Educational Research, 30(3), 3. 10.24452/sjer.30.3.4801

[bjep12741-bib-0074] Maaz, K. , Trautwein, U. , Lüdtke, O. , & Baumert, J. (2008). Educational transitions and differential learning environments: How explicit between‐school tracking contributes to social inequality in educational outcomes. Child Development Perspectives, 2(2), 99–106. 10.1111/j.1750-8606.2008.00048.x

[bjep12741-bib-0075] Marsh, H. W. (1987). The big‐fish‐little‐pond effect on academic self‐concept. Journal of Educational Psychology, 79(3), 283–295. 10.1037/0022-0663.79.3.280

[bjep12741-bib-0076] Marsh, H. W. , & Martin, A. J. (2011). Academic self‐concept and academic achievement: Relations and causal ordering. British Journal of Educational Psychology, 81(1), 59–77. 10.1348/000709910x503501 21391964

[bjep12741-bib-0077] Masters, G. N. (1982). A Rasch model for partial credit scoring. Psychometrika, 47(2), 149–174. 10.1007/BF02296272

[bjep12741-bib-0078] Mauthe, A. , & Rösner, E. (1998). Schulstruktur und Durchlässigkeit. Quantitative Entwicklungen im allgemeinbildenden weiterführenden Schulwesen und Mobilität zwischen den Bildungsgängen [School structure and permeability. Quantitative developments in the general secondary school system and mobility between educational pathways]. Jahrbuch der Schulentwicklung, 10, 87–126.

[bjep12741-bib-0079] Neuenschwander, M. , Gerber, M. , Frank, N. , & Rottermann, B. (2012). Übergang in die Sekundarstufe I. Schule und Beruf. VS Verlag für Sozialwissenschaften. 10.1007/978-3-531-94156-1_5

[bjep12741-bib-0080] Neugebauer, M. , & Schindler, S. (2012). Early transitions and tertiary enrolment: The cumulative impact of primary and secondary effects on entering university in Germany. Acta Sociologica, 55(1), 19–36.

[bjep12741-bib-0081] Neumann, I. , Duchhardt, C. , Grüßing, M. , Heinze, A. , Knopp, E. , & Ehmke, T. (2013). Modeling and assessing mathematical competence over the lifespan. Journal for Educational Research Online, 5(2), 80–109. 10.25656/01:8426

[bjep12741-bib-0082] Neumann, M. , Schnyder, I. , Trautwein, U. , Niggli, A. , Lüdtke, O. , & Cathomas, R. (2007). Schulformen als differenzielle Lernmilieus. Zeitschrift für Erziehungswissenschaft, 10(3), 399–420. 10.1007/s11618-007-0043-6

[bjep12741-bib-0083] Oakes, J. (2005). Keeping track: How schools structure inequality. Yale University Press.

[bjep12741-bib-0084] Ohle, A. , & Fischer, H. E. (2012). Physikalisches fachwissen von lehrkräften–ein vergleich zwischen grundschule, hauptschule und gymnasium [Physics knowledge of teachers‐A comparison between elementary school, secondary school and grammar school] (pp. 257–260). Bedingungen des Lehrens und Lernens in der Grundschule: Bilanz und Perspektiven.

[bjep12741-bib-0085] Ozer, M. , & Perc, M. (2020). Dreams and realities of school tracking and vocational education. Palgrave Communications, 6(1), 1–7. 10.1057/s41599-020-0409-4

[bjep12741-bib-0086] Pekrun, R. , vom Hofe, R. , Blum, W. , Götz, T. , Wartha, S. , & Jullien, S. (2006). Projekt zur analyze der Leistungsentwicklung in Mathematik (PALMA) [project to analyze the development of performance in mathematics]. In M. Prenzel & L. Allolio‐Näcke (Eds.), Untersuchungen zur Bildungsqualität von Schule (pp. 21–53). Abschlussbericht des DFG‐Schwerpunktprogramms.

[bjep12741-bib-0087] Pfost, M. , & Artelt, C. (2013). Reading literacy development in secondary school and effect of differential institutional learning environments. In M. Pfost , C. Artelt , & S. Weinert (Eds.), The development of reading literacy from early childhood to adolescence (pp. 229–277). University of Bamberg Press.

[bjep12741-bib-0088] Pfost, M. , Rausch, T. , Schiefer, I. M. , & Artelt, C. (2018). Zur Entwicklung von Gymnasiastinnen und Gymnasiasten ohne Gymnasialempfehlung on the development of grammar school students without a grammar school recommendation. Zeitschrift für Erziehungswissenschaft, 21(3), 511–534. 10.1007/s11618-017-0787-6

[bjep12741-bib-0089] Pishgar, F. , Greifer, N. , Leyrat, C. , & Stuart, E. (2021). MatchThem: Matching and weighting after multiple imputation. The R Journal, 13(2), 292–305. 10.32614/RJ-2021-073

[bjep12741-bib-0090] Pohl, S. , & Carstensen, C. H. (2013). Scaling of competence tests in the National Educational Panel Study—Many questions, some answers, and further challenges. Journal for Educational Research Online, 5(2), 189–216. 10.25656/01:8430

[bjep12741-bib-0091] Pribesh, S. , Gavigan, K. , & Dickinson, G. (2011). The access gap: Poverty and characteristics of school library media centers. The Library Quarterly, 81(2), 143–160. 10.1086/658868

[bjep12741-bib-0092] R Core Team . (2021). R: A language and environment for statistical computing. R Foundation for Statistical Computing. https://www.R‐project.org/

[bjep12741-bib-0093] Raghunathan, T. , Lepkowski, J. , Hoewyk, J. , & Solenberger, P. (2000). A multivariate technique for multiply imputing missing values using a sequence of regression models. Survey Methodology, 27(1), 85–95.

[bjep12741-bib-0094] Reichelt, M. , Collischon, M. , & Eberl, A. (2019). School tracking and its role in social reproduction: Reinforcing educational inheritance and the direct effects of social origin. The British Journal of Sociology, 70(4), 1323–1348. 10.1111/1468-4446.12655 30927272 PMC6767437

[bjep12741-bib-0095] Romer, P. M. (1990). Endogenous technological change. Journal of Political Economy, 98(5) Part 2, 71–102. 10.1086/261725

[bjep12741-bib-0096] Rosenbaum, P. R. , & Rubin, D. B. (1983). The central role of the propensity score in observational studies for causal effects. Biometrika, 70(1), 41–55. 10.1093/biomet/70.1.41

[bjep12741-bib-0097] Rost, J. (1996). Lehrbuch Testtheorie—Testkonstruktion [Textbook Test Theory‐Test Construction]. Verlag Hans Huber.

[bjep12741-bib-0098] Rubin, D. B. (2001). Using propensity scores to help design observational studies: Application to the tobacco litigation. Health Services and Outcomes Research Methodology, 2(3), 169–188. 10.1023/A:1020363010465

[bjep12741-bib-0099] Schafer, J. L. , & Graham, J. W. (2002). Missing data: Our view of the state of the art. Psychological Methods, 7(2), 147–177. 10.1037/1082-989X.7.2.147 12090408

[bjep12741-bib-0100] Scharenberg, K. (2014). Schule und Schulklasse als soziale Kontexte der Entwicklung im Jugendalter [school and school class as social contexts of development in adolescence]. KZfSS Kölner Zeitschrift für Soziologie Und Sozialpsychologie, 66, 317–348.

[bjep12741-bib-0101] Scheeren, L. (2022). The differential impact of educational tracking on SES gaps in educational achievement for boys and girls. European Sociological Review, 38(6), 942–958. 10.1093/esr/jcac012

[bjep12741-bib-0102] Schnittjer, I. , & Duchhardt, C. (2015). Mathematical competence: Framework and exemplary test items. Leibniz Institute for Educational Trajectories.

[bjep12741-bib-0103] Schnittjer, I. , & Gerken, A.‐L. (2018). Neps technical report for mathematics: Scaling results of starting cohort 2 for grade 2. (NEPS Survey Paper No. 47). Leibnitz Institute for Educational Trajectories. 10.5157/NEPS:SP47:1.0

[bjep12741-bib-0104] Shavit, Y. , & Müller, W. (2000). Vocational secondary education, tracking, and social stratification. In Y. Shavit & W. Müller (Eds.), Handbook of the sociology of education (pp. 437–452). Springer US.

[bjep12741-bib-0105] Slavin, R. E. (1990). Achievement effects of ability grouping in secondary schools: A best‐evidence synthesis. Review of Educational Research, 60(3), 471–499. 10.3102/00346543060003471

[bjep12741-bib-0106] Solga, H. , & Wagner, S. (2001). Paradoxie der Bildungsexpansion: Die doppelte Benachteiligung von Hauptschülern. Zeitschrift für Erziehungswissenschaft, 4, 107–127. 10.1007/s11618-001-0008-0

[bjep12741-bib-0107] Steinhauer, H. W. , Zinn, S. , Gaasch, C. , & Goßmann, S. (2016). NEPS technical report for weighting: Weighting the sample of grade 1 students of the national educational panel study (wave 1–3) (Vol. 66). NEPS Working Papers. Leibniz Institute for Educational Trajectories.

[bjep12741-bib-0108] Terrin, É. , & Triventi, M. (2023). The effect of school tracking on student achievement and inequality: A meta‐analysis. Review of Educational Research, 93(2), 236–274. 10.3102/00346543221100850

[bjep12741-bib-0109] Thys, S. (2018). The tertiary effect of social class: multilevel studies on the role of the primary school (teacher) in educational decision‐making (doctoral dissertation, Ghent University).

[bjep12741-bib-0110] Traini, C. , Kleinert, C. , & Bittmann, F. (2021). How does exposure to a different school track influence learning progress? Explaining scissor effects by track in Germany. Research in Social Stratification and Mobility, 76, 100625. 10.1016/j.rssm.2021.100625

[bjep12741-bib-0111] Valtin, R. (2005). Länger gemeinsam lernen—eine notwendige, aber nicht hinreichende bildungspolitische Forderung [Learning together for longer‐a necessary but not sufficient educational policy demand]. In G.‐B. Reinert (Ed.), Bildungsreform als Lebensreform (pp. 243–251). Peter Lang.

[bjep12741-bib-0112] van Ewijk, R. , & Sleegers, P. (2010). The effect of peer socioeconomic status on student achievement: A meta‐analysis. Educational Research Review, 5(2), 134–150. 10.1016/j.edurev.2010.02.001

[bjep12741-bib-0113] Warm, T. A. (1989). Weighted likelihood estimation of ability in item response theory. Psychometrika, 54(3), 427–450. 10.1007/BF02294627

[bjep12741-bib-0114] van de Werfhorst, H. G. , & Mijs, J. J. (2010). Achievement inequality and the institutional structure of educational systems: A comparative perspective. Annual Review of Sociology, 36, 407–428.

[bjep12741-bib-0115] Zinn, S. , Würbach, A. , Steinhauer, H. W. , & Hammon, A. (2020). Attrition and selectivity of the NEPS starting cohorts: An overview of the past 8 years. AStA Wirtschafts‐ Und Sozialstatistisches Archiv, 14(2), 163–206. 10.1007/s11943-020-00268-7

